# Illuminating the pathway for the next generation of cardiovascular medicine practitioners and researchers: Highlights of the Joint PASCAR–SCC clinical symposium on hypertension and heart failure, Cameroon

**Published:** 2017

**Authors:** Martin H Abanda, Anastase Dzudie, Loryane Nganhyim, Bonaventure S Dzekem, Anastase Dzudie, Ba Hamadou, Henry Luma, Marie Solange Douala, Eugene Belley Priso, Anastase Dzudie, Yves Monkam, Henry Luma, Marie Solange Douala, Theophile N Nana, Eugene Belley Priso, Anastase Dzudie, George Nel, Ana O Mocumbi, Karen Sliwa, Anastase Dzudie, Ba Hamadou, Yves Monkam, Ana O Mocumbi, Simon Stewart, Karen Sliwa

**Affiliations:** Clinical Research Education, Networking and Consultancy (CRENC), Douala, Cameroon; Clinical Research Education, Networking and Consultancy (CRENC), Douala, Cameroon; Clinical Research Education, Networking and Consultancy (CRENC), Douala, Cameroon; Clinical Research Education, Networking and Consultancy (CRENC), Douala, Cameroon; Faculty of Medicine and Biomedical Sciences, University of Yaoundé 1, Cameroon; Faculty of Medicine and Biomedical Sciences, University of Yaoundé 1, Cameroon; Faculty of Medicine and Biomedical Sciences, University of Yaoundé 1, Cameroon; Faculty of Medicine and Biomedical Sciences, University of Yaoundé 1, Cameroon; Faculty of Medicine and Biomedical Sciences, University of Yaoundé 1, Cameroon; Douala General Hospital, Douala, Cameroon; Douala General Hospital, Douala, Cameroon; Douala General Hospital, Douala, Cameroon; Douala General Hospital, Douala, Cameroon; Douala General Hospital, Douala, Cameroon; Douala General Hospital, Douala, Cameroon; Pan-African Society of Cardiology; Pan-African Society of Cardiology; Pan-African Society of Cardiology; Pan-African Society of Cardiology; Cameroon Cardiac Society; Cameroon Cardiac Society; Cameroon Cardiac Society; Instituto Nacional de Saúde, and Eduardo Mondlane University, Maputo, Mozambique; Mary Mackillop Institute of Health Research, Australian Catholic University, Australia; Hatter Institute of Cardiovascular Research, University of Cape Town, Cape Town, South Africa

## Abstract

The Pan-African Society of Cardiology roadmap aims to achieve a 25% control of hypertension by the year 2025. Whether this is attainable or not depends largely on the capacity of healthcare providers and policy makers to address the rising prevalence of hypertension and its complications, including heart failure. Task sharing is fundamental in optimising hypertension control.

The Clinical Research Education, Networking and Consultancy (CRENC) engaged with the Pan-African Society of Cardiology (PASCAR) and the Cameroon Cardiac Society (SCC) in a joint hypertension and heart failure symposium at the Douala General Hospital in 2016. The primary aims were to foster clinical research in cardiovascular medicine by raising awareness on cardiovascular diseases, to provide evidence-based training of an international standard, to encourage the conduction and dissemination of high-quality research, and to build programmes for continuing medical education. The secondary aim was to potentiate the 2nd Douala Research and Scientific Days.

The symposium, which featured didactic lectures interspaced with oral/poster abstract presentations and a clinical visit, culminated in the launching of the book Heart of Africa, and the Young Investigator award. It is hoped that these served to capacitate existing cardiovascular structures, breed the next generation of cardiovascular physicians and researchers, and imprint a trail of clinical research excellence to be emulated in Cameroon and beyond.

## Introduction

Sub-Saharan Africa bears about 80% of the global burden of cardiovascular disease (CVD).^1^,^2^ As the leading continental cardiovascular society, the Pan-African Society of Cardiology (PASCAR) has identified hypertension as the key area of priority action to reduce the burden of CVD in Africa. Shifting paradigms through task sharing has been highlighted among the 10 pillars to beat hypertension in Africa.^3^ Aligned with these strategies, the Clinical Research Education, Networking and Consultancy (CRENC) engaged with PASCAR at the Joint PASCAR–SCC seminar on hypertension and heart failure at the Douala General Hospital (11–12 October 2016), supported by the National Institutes of Health (NIH) Fogarty International Center (Advancing Science for Global Health).

Over 300 participants, including experienced, mid and early medical scientists, both national and international, were invited to discuss the science of hypertension and heart failure. This consisted of didactic lectures, abstracts (10 oral, 12 posters), a clinical visit and more than 50 oral communications for a target audience of about 300 participants.

International faculty consisted of experts from the PASCAR task force on hypertension and heart failure and the Mary McKillop Institute for Health Research: Center for Research Excellence for reducing inequalities in cardiovascular disease burden at the Australian Catholic University (ACU). These experts were Profs Karen Sliwa, Simon Stewart and Ana O Mocumbi, Drs Dike Ojji and Kemi Tibazarwa, George Nel and Ms Ashley Kimberley Keates.

## Pre-meeting activities: (9 October: pacemaker marathon; 10 October: mass media conference)

These activities were aimed at increasing public and policy makers’ awareness on cardiac disease in general, as well as narrowing the gap between pacemaker needs and safety, and the current status of pacemaker implantation in Cameroon. There was a pacemaker marathon involving people with implanted pacemakers under close monitoring, and the launching of the ‘Pace4life Cameroon’ project.

## Day 1: Joint PASCAR–SCC clinical symposium on hypertension and heart failure

The opening ceremony was chaired by Dr Yves Monkam who offered a word of welcome to all participants and highlighted the importance of such an event for the advancement of medicine and research. He also commended young physician-researchers whose abstracts were accepted for presentation.

The first part of this symposium was the PASCAR and Medtronic pacing and heart-failure training session, which was chaired by Drs Félicité Kamdem, Yves Monkam, Archange Nzali and Anastase Dzudie. Dr Loryane Nganhyim (CRENC affiliate) presented the LOng-TErm Prognosis of Patients with clinical indication for a cardiac Pacemaker Implantation (LOTEPPI) study, in which only about one in two patients had access to cardiac pacemakers but three in four patients with cardiac pacemakers had improved quality of life and likelihood of survival over three years. Re-using recycled cardiac pacemakers was suggested as an affordable alternative to new pacemakers in resource-limited settings such as Cameroon.

Ms Kimberley Keates (Australia) gave a talk on the epidemiology of CVD in Africa. She highlighted that CVDs are common and that all regions in Africa are affected. However a major challenge remains the paucity of data, which limits detailed analyses of the spectrum and burden of CVD. Prof K Sliwa encouraged the publication of all studies conducted, as this will allow the possibility of future use of data, as well as a better or more realistic contextual analysis of CVD in Africa and Cameroon in particular. She also discussed the findings of the HOPE III trial.^4^-^6^

The second part was the hypertension and heart failure symposium, chaired by Emeritus Prof Walinjom FT Muna (Cameroon), Prof Simon Stewart (Australia), Prof Karen Sliwa (South Africa) and Dr Yves Monkam (Cameroon). Dr Monkam offered a word of welcome to international faculty, especially from PASCAR, on behalf of the Cameroon Cardiac Society.

This was followed by Dr Biholong’s presentation ‘Management of mild–moderate hypertension’,^7^ and Dr Ba Ahmadou’s presentation on new recommendations from the European Society of Cardiology (ESC) on the treatment of heart failure in 2016.^8^ Dr Dzudie presented ‘What every general practitioner should know regarding the management of hypertension’ and he enumerated and explained the PASCAR 10 pillars to beat hypertension in Africa.^3^

The final part of this session was the NIH Fogarty non-communicable diseases leadership activity. Mr Ferdinant Mbidzenyuy presented ‘How to foster a research culture in limited-resource settings: the case of Cameroon Baptist Convention’. This was followed by Prof Mocumbi’s presentation ‘Building and maintaining a scientific reputation’. The talk was drawn from her own experience in Mozambique. She laid emphasis on the mentor–mentee system as one of the factors that facilitate building and maintaining a scientific reputation.

Dr Dzudie offered concluding remarks for day one. He highlighted the burden of CVDs in Africa, the antihypertensive effect of statins (Hope III trial), cardiovascular risk-factor stratification, the role of the general practitioner in the management of hypertension, being open to the mentor– mentee system, and the dissemination of research findings. He rendered tributes to the organisers and partners, both local and international, and gratitude to all participants.

## Day 2: Grand rounds: cardiovascular diseases in pregnancy

A clinical visit to the obstetrics and gynaecology ward of the Douala General Hospital was conducted. This was led by Profs Karen Sliwa and Eugene Belley Priso, among a delegation of about 40 clinicians. Three cases were identified on the ward and discussed: pregnancy and cardiac pacemakers, peripartum cardiomyopathy and postpartum cardiomyopathy. This activity took place in parallel with a scientific presentation of eight abstracts by early-career scientists.

After the ‘grand rounds’, there was a symposium on cardiovascular disease in pregnancy, chaired by Profs Sliwa, Mocumbi and Priso. A most memorable presentation on cardiovascular diseases in pregnancy was given by Prof Sliwa. The burden of cardiac disease in pregnancy, common patterns of presentation, major signs and symptoms, and contributing factors were enumerated. This was followed by a presentation from Prof Mocumbi ‘When should women with cardiac disease in pregnancy give birth next?’ There were also presentations from Prof Mboudou and Dr Balla on the challenges of cardiac disease in pregnancy in Cameroon, and the management of diabetes in pregnancy, respectively.

## Launching the Heart of Africa

The book titled Heart of Africa was unveiled for the first time in Cameroon and senior editors present were Profs Sliwa, Stewart and Mocumbi, as well as associate editor Ms Kimberly Keates.^9^ Prof Sliwa presented all the authors with a copy of the book. Some of the authors present at the time were Drs Kemi Tibazarwa, Dike Ojji and Anastase Dzudie.

**Fig. 1.**
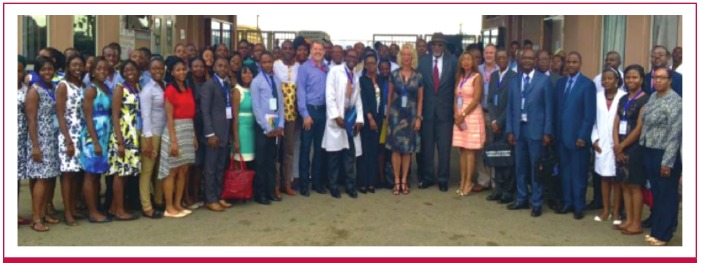
Group picture, PASCAR symposium on hypertension and heart failure.

## National Institute of Health Fogarty Young Researcher awards

All oral/poster abstract presentations were evaluated on scientific merit (60%) and the quality of the oral/poster presentation (40%). The Heart of Africa was awarded to young investigators for the best abstract presentation. Of 20 oral/abstract presentations evaluated, the top three rated abstracts and presenters were awarded the Young Investigator award. Winners in order of merit were Dr Martin Abanda, Mr Ferdinant Mbidzenyuy and Dr Essama.

## Conclusion

The high burden of CVD warrants clinical research priorities such as the need for proper record archiving, dissemination of research findings, and preventative approaches to CVD. Emerging diseases such as cardiac disease in pregnancy also deserves strategies such as the establishment of good clinical registries. Bridging the gap between specialists and general practitioners will serve to improve the quantity and quality of healthcare delivery and by extension, the potential attainment of the 25 × 25 World Health Organisation objective for cardiovascular diseases.
